# Colorimetric detection of melamine in milk by using gold nanoparticles-based LSPR via optical fibers

**DOI:** 10.1371/journal.pone.0177131

**Published:** 2017-05-05

**Authors:** Keke Chang, Shun Wang, Hao Zhang, Qingqian Guo, Xinran Hu, Zhili Lin, Haifeng Sun, Min Jiang, Jiandong Hu

**Affiliations:** 1 College of Mechanical and Electrical Engineering, Henan Agricultural University, Zhengzhou, China; 2 State Key Laboratory of Wheat and Maize Crop Science, Zhengzhou, China; 3 School of Human Nutrition and Dietetics, McGill University, Macdonald Campus, Ste Anne de Bellevue, Quebec, Canada; 4 College of Information Science and Engineering, Huaqiao University, Xiamen, China; 5 College of life sciences, Henan Agricultural University, Zhengzhou, China; Institute of Materials Science, GERMANY

## Abstract

A biosensing system with optical fibers is proposed for the colorimetric detection of melamine in liquid milk samples by using the localized surface plasmon resonance (LSPR) of unmodified gold nanoparticles (AuNPs). The biosensing system consists of a broadband light source that covers the spectral range from 200 nm to 1700 nm, an optical attenuator, three types of 600 μm premium optical fibers with SMA905 connectors and a miniature spectrometer with a linear charge coupled device (CCD) array. The biosensing system with optical fibers is low-cost, simple and is well-proven for the detection of melamine. Its working principle is based on the color changes of AuNPs solution from wine-red to blue due to the inter-particle coupling effect that causes the shifts of wavelength and absorbance in LSPR band after the to-be-measured melamine samples were added. Under the optimized conditions, the detection response of the LSPR biosensing system was found to be linear in melamine detection in the concentration range from 0μM to 0.9 μM with a correlation coefficient (R^2^) 0.99 and a detection limit 33 nM. The experimental results obtained from the established LSPR biosensing system in the actual detection of melamine concentration in liquid milk samples show that this technique is highly specific and sensitive and would have a huge application prospects.

## Introduction

Melamine (C3H6N6; molecular weight: 126.12) is an organic compound with a 1, 3, 5-triazine and 2, 4, 6-triamine skeleton and the aqueous solution of melamine is weakly alkaline. Generally, melamine is often a basic chemical that can be used to make, for example, fire retardant, water-reducing agent and formaldehyde cleaner. Due to its biotoxicity, excessive intake of melamine will cause great harm to human health and lead to diseased kidney or urinary system [1, 2]. Melamine is therefore not approved by governments as a food additive in animal feed or human food. However, melamine was sometimes illegally added to protein-rich food by unethical manufactures to increase nitrogen level (66% nitrogen by mass). The most famous food safety accidents related to melamine occurred in China in 2008. Melamine was illegally added into the infant milk powder by a dairy company that caused about 40,000 children affected with diseases. As reported in 2012, international experts in Europe and USA have limited the melamine level in food at the concentration less than 2.5 mg/kg [3]. Thus there is an increasing demand for effective and reliable methods proposed to detect the concentration of melamine in food.

In recent years, a growing body of researches has focused on the detection of melamine in food and drinking water. The most commonly used methods for melamine detection are the chromatographic analysis methods, including high performance liquid chromatography (HPLC) [4], mass spectrometry (MS) [5], gas chromatography/mass spectrometry (GC/MS) [6], high performance liquid chromatography/mass spectrometry (HPLC/MS) [7], capillary zone electrophoresis/mass spectrometry [8], and electrochemical methods [9,10]. Enzyme-linked immunosorbent assay (ELISA), surface enhanced Raman spectroscopy (SERS), two-photon photoluminescence, dynamic light scattering, hyper-rayleigh scattering, and visible and near-infrared bulk optical properties of raw milk have also been utilized for the detection of melamine [11–15]. However, most of these techniques have the disadvantages such as complicated pre-treatment, time-consuming steps and high-cost instruments, which cannot meet the requirement for rapid detection. Hence, there is an urgent need to develop a fast, low-cost, highly sensitive and selective biosensing system to determine the trace melamine in food samples. Recently, AuNPs-based colorimetric sensors have attracted great attention due to the unique properties of AuNPs, such as the excellent optical property, electronic property, biocompatibility, stability and high extinction coefficients [16–18]. Various standard protocols and recent advances for the shape-controlled synthesis of nanocrystals have been reported [19]. Several studies have already been conducted for the colorimetric detection of melamine in milk based on AuNPs localized surface plasmon resonance (LSPR) that offers an easy and rapid way for melamine detection [20–23]. The influence produced by the size and shape of AuNPs in LSPR biosensing system has been investigated [24–28]. Gold nanostars immobilized onto flexible PDMS substrates via a simple solution process based on electrostatic self-assembly have been demonstrated [29, 30]. However, most of the colorimetric detection methods rely on an ultraviolet-visible (UV-Vis) spectrophotometer, which is expensive and not portable.

In this paper, we report a localized surface plasmon resonance (LSPR) biosensing system that utilizes AuNPs for the colorimetric detection of melamine in liquid milk. When the AuNPs solutions are with different melamine concentrations, the absorbance ratios of light at wavelength 520 nm are different due to the AuNPs changed from dispersion to aggregation. Based on this phenomenon, a standard model is established to detect the melamine concentrations in liquid milk samples and the melamine at low concentration can be detected rapidly and sensitively by the proposed LSPR biosensing system with optical fibers.

## Principle of LSPR and the experimental setup

**Fig 1:**
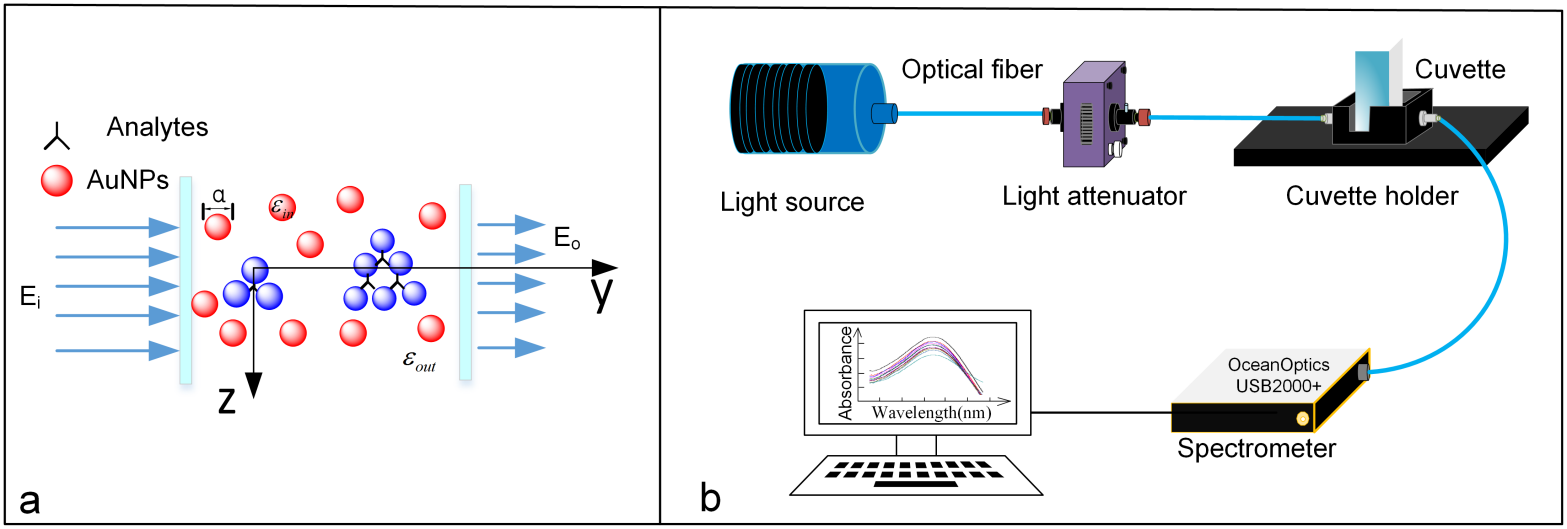
Schematic diagrams illustrating the principle of (a) localized surface plasmon resonance and (b) the optical fiber-based LSPR biosensing system.

The nanoparticle-based optical sensing techniques have been found to be effective for quantitative detection of chemical and biological targets. The principle of this method is based on the analysis of LSPR spectrum of noble metal nanoparticles, which is pertaining to the refractive index changes caused by the surrounding environment (see Fig 1a). In considering a spherical nanoparticle of radius *a* and irradiated by a light at wavelength *λ* (*a* is much smaller than the wavelength of *λ*), the LSPR resonance occurs when the frequency of incident electromagnetic wave is coincident with the oscillation frequency of the oscillating electrons on the surface of nanoparticle. The extinction spectrum of the gold sphere can be calculated by solving Maxwell’s equations using the quasi-static approximation [31]:

$$E(\lambda )=\frac{24\pi^{2}N\times a^{3}\times \varepsilon_{out}^{3/2}}{\lambda \times \text{ln}(10)}[\frac{\varepsilon_{i}(\lambda )}{{\left(\varepsilon_{i}(\lambda )+\chi \times \varepsilon_{out}\right)}^{2}+\varepsilon_{i}{\left(\lambda \right)}^{2}}].$$ (1)

Where *ε*_*in*_ and *ε*_*out*_ are the dielectric constants of the gold nanoparticle and the external environment, respectively. Since the gold metal is dispersive, the complex dielectric constant depends on the wavelength of incident light, *λ*, and can be expressed by *ε*(*λ*) = *ε*_*r*_(*λ*) + *ε*_*i*_(λ), where *ε*_*r*_(*λ*) and *ε*_*i*_(*λ*) represent the real and imaginary parts, respectively. *N* is the electron density. χ is the shape factor of the metal nanoparticle for a typical value of 2 for the case of a sphere.

The occurrence of LSPR depends on the size, shape, interparticle space and dielectric properties of the material, as well as the dielectric properties of the surrounding environment. The maximum wavelength (*λ*_max_) of LSPR extinction for nanoparticles is highly sensitive to the refractive index *n* and can be measured by using ultraviolet-visible extinction spectroscopy. Therefore, *λ*_max_ will shift when the adsorbate is bound to AuNPs due to the change of refractive index of local environment. The LSPR shift is mainly attributed to the adsorbate and the shift of LSPR extinction wavelength maximum Δ*λ*_max_ is given as follows:

$$\Delta \lambda_{\text{max}}=m\times \Delta n\times [1-\text{exp}(-\frac{2d}{l_{d}})].$$ (2)

where *m* is the bulk refractive-index response of the nanoparticle, Δ*n* is the change of refractive index induced by the adsorbate, *d* is the effective adsorbate layer thickness, and *l*_*d*_ is the electromagnetic field decay length (approximated as an exponential decay) [32,33]. In theory, the extinction spectrum is determined by both the absorption and scattering characteristics of AuNPs and obtained by measuring the ratio of light transmitting through the AuNPs solution to the incidence light. In this work, optical fibers and spectrometer are utilized to record the transmission ultraviolet-visible spectra in a straightforward mode, as shown in Fig 1(b). Since the diameters of AuNPs are usually less than 10 nm and the concentration of AuNPs is very low in the solution, the scattering effect of AuNPs is negligible as compared with the absorption effect. Thus, the absorbance can be approximatively calculated by the Lambert-Beer’s law.

The schematic diagram of the optical fiber-based LSPR detection system is depicted In Fig 1(b), where the tungsten halogen lamp is used as the broadband light source with spectrum ranging from 200nm to 1700 nm and the optical attenuator is used to adjust and optimize the intensity of light source that was delivered to the cuvette for spectral measurement. The light from the source is transmitting through an optical fiber of diameter 600 μm before entering the cuvette filled with sample solution where the interaction between the sample molecules and the incident light photons occurs. After that, the transmitted light enters the spectrometer through another optical fiber and the output light is converted into electrical signals via the CCD detector of spectrometer. The values of light absorbance can be calculated by the Beer-Lambert Law, $A(\lambda )={\text{log}}_{10}(\frac{I_{R}(\lambda )-I_{B}(\lambda )}{I_{S}(\lambda )-I_{B}(\lambda )})$, where *I*_*R*_ is the reference signal intensity, *I*_*B*_ is the background signal intensity (or dark noise) and *I*_*S*_ is the sample signal intensity.

## Experimental results and discussion

### Reagents

Chloroauric acid (HAuCl_4_), sodium citrate, and sodium carbonate (Na_2_CO_3_) were purchased from Sinopharm Chemical Reagent Co., Ltd. (Shanghai, China). Glucose, fructose and lactose were purchased from Tianjin Chemical Co., Ltd. Melamine was obtained from Tianjin Reagent Chemical Reagent Co., Ltd. (Tianjin, China). Liquid milk was purchased from the local supermarket. All solvents and reagents were of analytical grade and were used without further purification. Ultrapure water was used throughout the whole experiment. All glassware used in the experiment were soaked in nitrohydrochloric acid and rinsed thoroughly in water and dried in air before use.

### Preparation of AuNPs

The AuNPs used in the experiments were prepared by the reduction of chloroauric acid using sodium citrate. 1 mL of sodium citrate (1%) was rapidly injected into 0.01% HAuCl_4_ (100 *mL*) boiling aqueous solution and further refluxed for 15 min. The mixture was then cooled with distilled water and continuously stirred before its temperature returned to room temperature. The formed wine-red AuNPs solution was then stored at 4°*C* for further use. According to the measured UV-visible spectra of AuNPs solution obtained from this preparation, it is found that the maximum absorption wavelength of AuNPs is 520 nm. Moreover, it also can be inferred that the sizes of AuNPs were about 13 nm from the transmission electron microscopy (TEM) images [34].

### Establishment of detection method

Firstly, 100 μL of AuNPs solution was taken using a centrifugal tube (full scale, 500 μL) and diluted with 300 μL of ultrapure water. Then, 100 μL of melamine solutions (standard level) with different concentrations were poured into the AuNPs solutions to form the sample solutions. These sample solutions were evenly mixed by the Vortex-Qilinbeier 5 vibrator before being individually transferred to the cuvette. After 30 min reaction time, the absorption spectrum was recorded continuously every 15s and 120 sets of spectra data were stored totally. Then the calibration curve was established according to the relationship between the absorbance at the maximum absorption wavelength 520 nm and the concentrations of melamine solution.

### Pretreatment of liquid milk samples

Firstly, 1.2 mL of 10% trichloroacetic acid and 1.5 mL of chloroform were added into 4 mL of liquid milk. The mixture was placed into the centrifugal tube and agitated for 2 min with the vibrator before being placed into an ultrasonic bath for 15 min to reach equilibrium. After that, the liquid milk suspensions were homogenized and then centrifuged for 10 min at a speed of 13000 rpm. The clear supernatant was taken into another centrifugal tube and the 1.0 mol/L Na_2_CO_3_ solution was added to adjust the pH to 4.3~4.5. Again, this supernatant was centrifuged for 3 min at a speed of 3000 rpm. Finally, the supernatant was stored in the refrigerator for further use.

## Results and discussion

### Melamine measurement based on colorimetric detection

**Fig 2:**
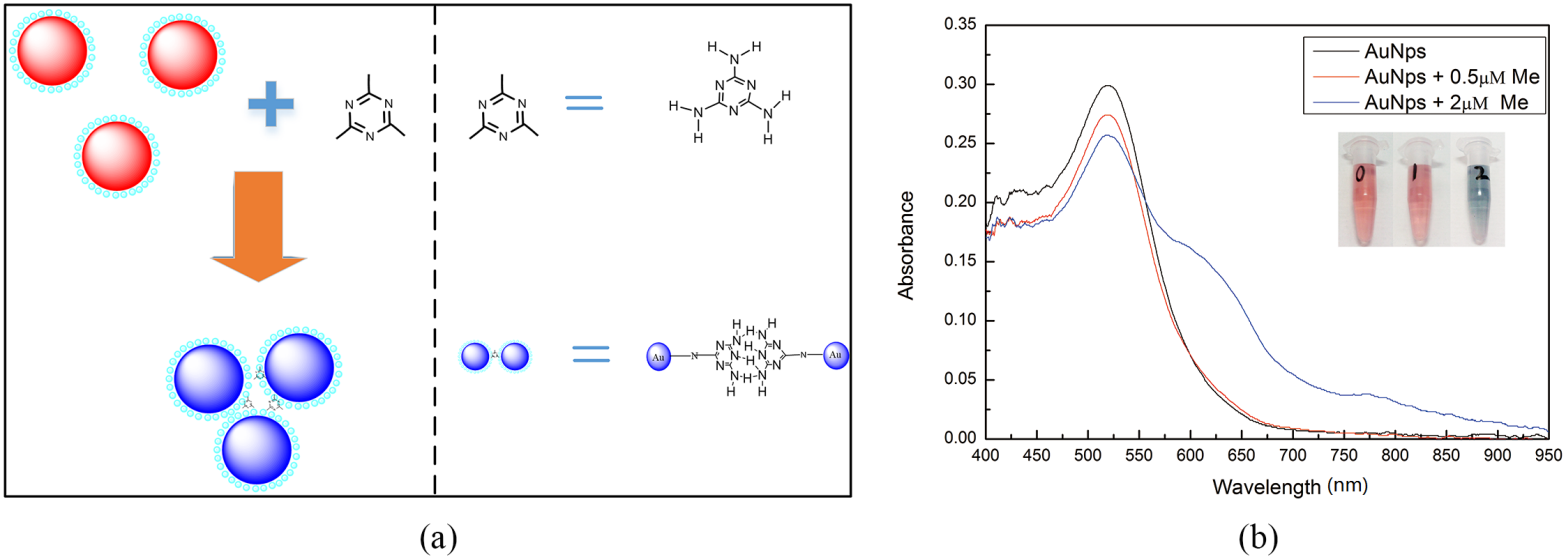
(a) Mechanism of the colorimetric detection of melamine based on the unmodified AuNPs; (b) UV-Visible absorption spectra of AuNPs (blank), AuNPs+0.5μM melamine and AuNPs+2μM melamine.

The mechanism for the colorimetric detection of melamine is illustrated in Fig 2(a). The synthesized AuNPs are stable in aqueous solution due to the repulsion force between citrate anions on the surface of AuNPs which prevents the AuNPs from aggregating. The citrate-stabilized AuNPs solution shows a wine-red color. When the melamine solution is added, the electrostatic repulsion between AuNPs is screened, which leads to the aggregation of AuNPs and causes the solution color to turn blue. The reason is that three amine groups (-NH_2_) of melamine molecules would interact with the AuNPs and the dissociation of citrates ions from the surface of AuNPs makes them losing the ability to stabilize AuNPs.

In order to further validate the above detection mechanism, a series of UV-Visible spectra of AuNPs are measured by using the optical fiber-based LSPR detection system under different experimental conditions. When the melamine is added into the AuNPs solution, the induced aggregation effect makes the localized surface plasmon resonance (LSPR) absorption band of AuNPs red-shifted and broadened. As shown in Fig 2(b), the absorption spectra of AuNPs solution have maximum absorption peaks at 520 nm. The maximum absorption peak of the solution decreases when the added melamine is with a concentration of 0.5 μM. Further, when 2 μM melamine is added into the AuNPs solution, a new absorption peak at about 640 nm appears. From the inset of Fig 2(b), it can be seen clearly that the color of AuNPs solution changed from red to blue. The UV-Visible absorption spectra of AuNPs (Blank), AuNPs+0.5μM melamine, AuNPs+2μM melamine and AuNPs+10μM melamine were shown in S1 Fig.

**Fig 3:**
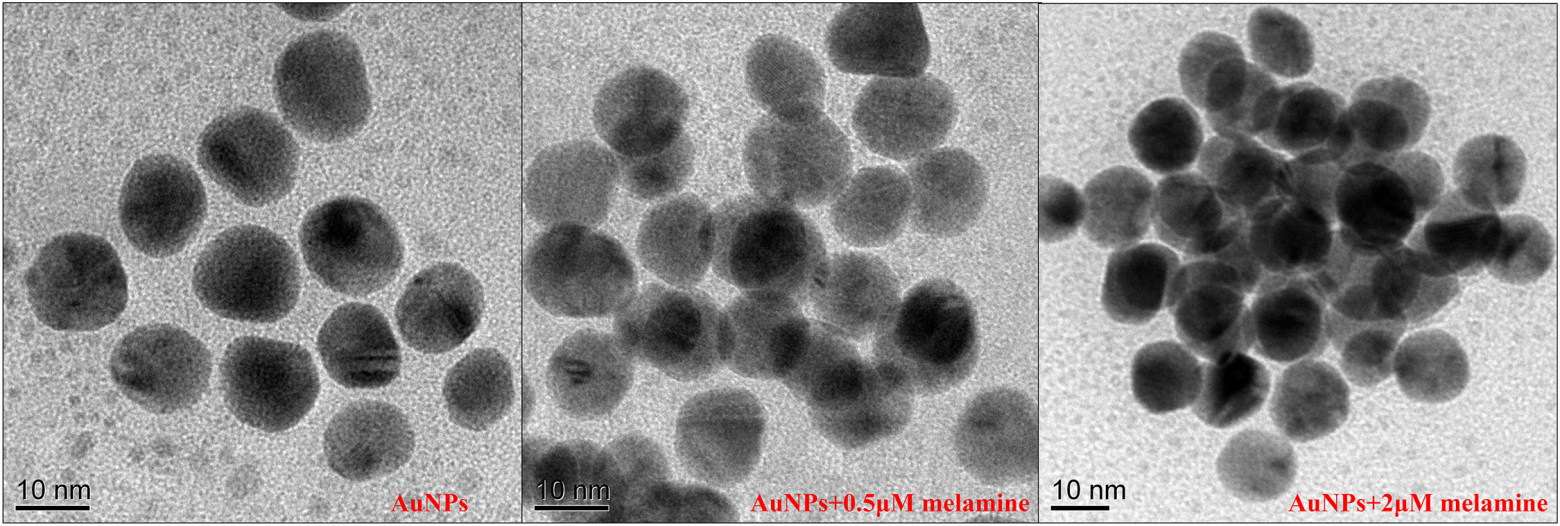
TEM images of the citrate-stabilized AuNPs, AuNPs+0.5μM melamine and AuNPs+2μM melamine.

The TEM images shown in Fig 3 have corroborated the actual sizes and aggregation effect of AuNPs, where the bare AuNPs are dispersed and the aggregation effect occurs in the presence of melamine. Moreover, the aggregation effect became more obvious with the increased concentration of melamine.

### Establishment of standard curve

**Fig 4:**
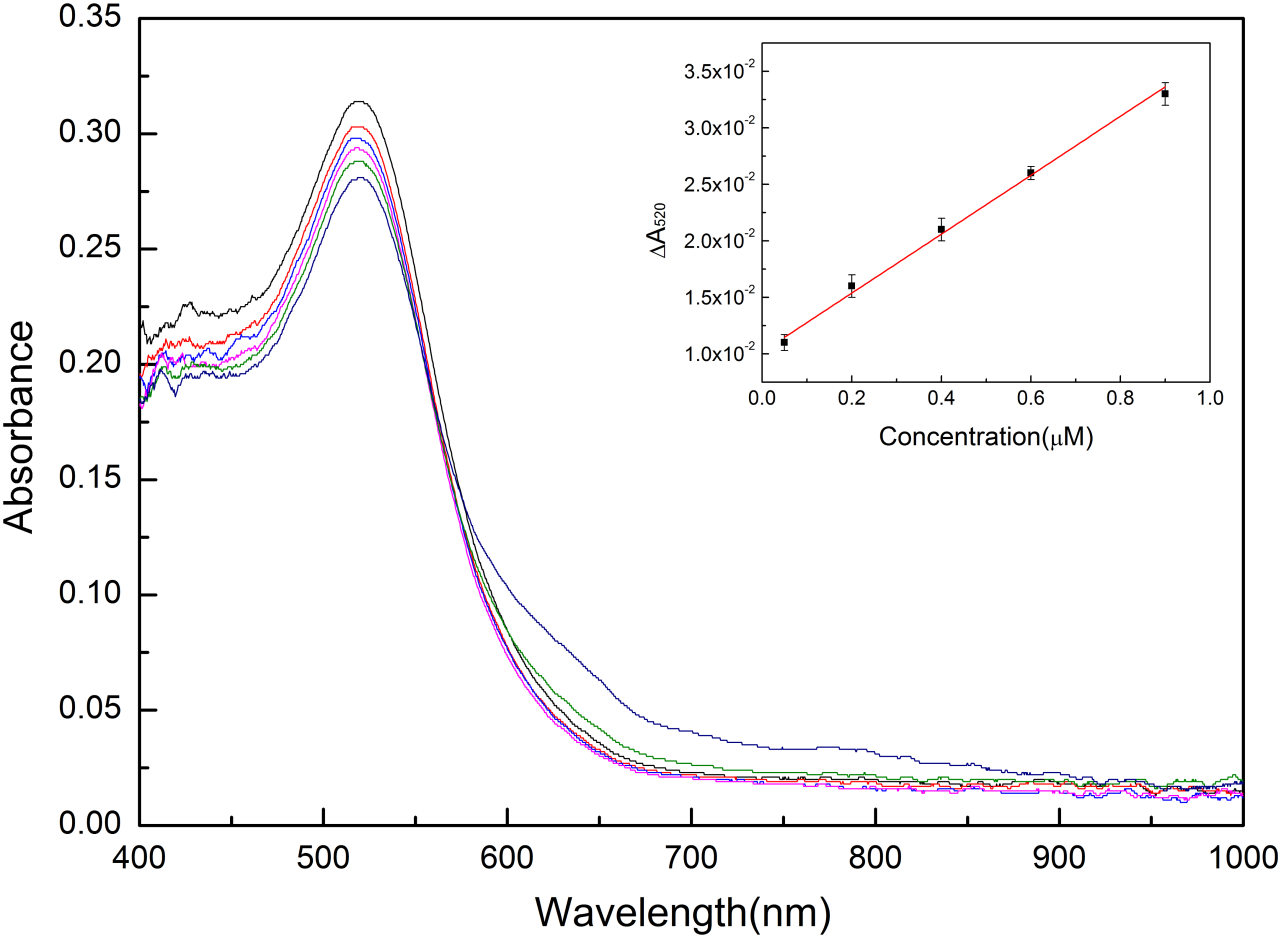
UV-Visible absorption spectra of AuNPs added with different concentrations of melamine (0, 0.05, 0.2, 0.4, 0.6, and 0.9 μM) and the calibration curve of melamine detection (inset).

The standard melamine solutions with different concentrations, 0.05, 0.2, 0.4, 0.6 and 0.9 μM, are added into the AuNPs solutions, respectively. As can be seen from Fig 4, the increment of melamine concentration leads to the decreasing absorbance of AuNPs solutions at wavelength 520nm. In order to quantitatively study the relationship between the melamine concentration and the AuNPs absorbance, we set the absorbance difference (Δ*A*) between the bare AuNPs solution and the AuNPs solutions added with melamine of different concentrations as the abscissa variable and the melamine concentration as the ordinate variable. Then a good linear curve is obtained with an R square of 0.99 between the concentration and absorbance. The fitting equation of the linear curve is *y* = 0.026*x* + 0.01 and the detection limit is 33 nM as calculated according to the formula $\frac{3\alpha }{slope}$, where *α* is the standard deviation and *slope* is the slope of the fitting straight line. The obtained linear calibration curve is shown in the inset of Fig 4.

As compared with some other methods with the detection limits shown in Table 1, the optical fiber-based LSPR method for melamine detection has a high sensitivity. Furthermore, from Table 1, the linear relation between the absorbance ratio and the concentration of melamine in the range of 2.0–250.0 μM [35], a linear relation between the absorbance and the melamine concentration in the range of 0.4–4μM [36], a linear correlation between the absorbance and the melamine concentration ranging from 0.9μM to 3.1μM [37] have been validated. Therefore, it can be seen that the proposed method not only provides a sensitive colorimetric detection of melamine at a low concentration in the range of 0~0.9 μM with a correlation coefficient (R2) of 0.99 and a detection limit of 33 nM, but also makes a compact and inexpensive spectroscopic instrumentation possible.

**Table 1:** Comparison of the detection limits of the various methods in the colorimetric detection of melamine in milk.

Techniques	Detection limit	References
Colorimetric method based on AgNPs	2.32μM	Ping et al., Food Control (2012) [35]
Colorimetric method based on Mb—mediated AuNPs	0.238 μM	Xin et al., Food Chemistry (2015) [36]
Colorimetric method based on SAA—AgNPs	10.6 nM	Song et al., Food Control (2015) [37]
Optical fiber -based localized surface plasmon resonance of AuNPs (this work)	33 nM	

### Experiment of specificity

**Fig 5:**
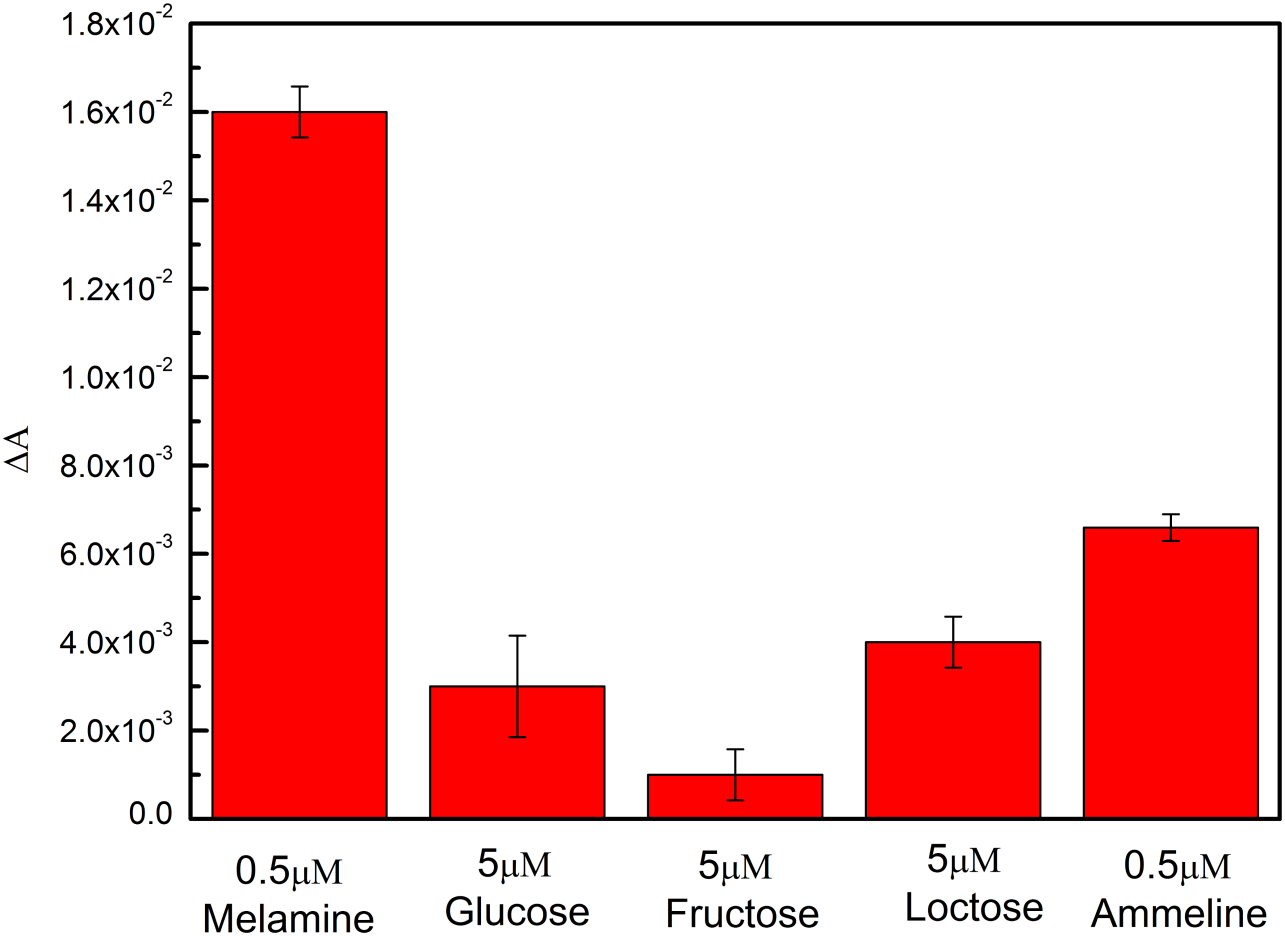
Specificity of the AuNPs for melamine detection, compared with other interfering compounds in liquid milk.

In order to investigate the specificity of this method for the detection of melamine concentrations in liquid milk samples, several interfering compounds commonly present in liquid milk, such as glucose, fructose, lactose and ammeline (C3H5N5O), which is the hydrolysis product of melamine, were chosen for the absorption spectra experiment as the same to those of melamine. The specificity study was estimated by measuring the absorption difference Δ*A* in the presence of melamine and other interfering compounds. During the experiment of specificity, the concentration of standard melamine solution was 0.5 μM, while the interfering compounds are with a much high concentration, 5 μM. As shown in Fig 5, the absorption difference Δ*A* from the melamine measurement is more than ten times as big as the effect from saccharide (the interfering compounds in milk), however, it is less than twice the absorption difference Δ*A* from ammeline. The phenomenon demonstrates that the saccharides do not interfere the melamine detection. The results imply that the presence of these interfering compounds cannot cause much aggregation effect to AuNPs and hasn't much impact on the melamine detection by using this optical fiber-based LSPR biosensing system.

### Detection of melamine in liquid milk samples

Further experiments are conducted in this section to validate the feasibility of the proposed method for detecting melamine in real milk samples mixed with different amounts of melamine. The milk samples with different melamine concentrations are prepared following the procedure described above in Section 4.2. The melamine solutions with concentrations 0.5 μM, 1.5 μM and 2.5 μM were agitated for 0.5 min with the Vortex-Qilinbeier5 vibrator, respectively. Precisely, 100 μL of melamine solutions was taken separately from above 0.5 μM, 1.5 μM and 2.5 μM of melamine solutions, and then 400 μL of AuNPs was added to reach the five-fold dilution. Afterwards, 100 μL of the mixtures were taken and their absorption spectra are measured by using this optical fiber-based LSPR detection system. The experiment results show that response relationship between the difference of absorbance values at wavelength 520 nm and the melamine concentrations of the liquid milks is still linear in the concentration range from 0.1μM to 0.9 μM. The calculated recovery rate of the colorimetric detection of melamine samples according to the measurement results is shown in Table 2. A recovery rate of 99.2%~111% was obtained with the detection limit of 33 nM. This recovery experiment shows that the optical fiber-based localized surface plasmon resonance of AuNPs for the colorimetric detection of melamine in liquid milk is also suitable for the rapid melamine detection in practice.

**Table 2:** The obtained recovery rate from measurement results.

Number of Measurement	Concentration of melamine (μM)		(%) Recovery (n = 3)
	Spiked concentration	Detected concentration (Mean^1^+RSD^2^)	
1	0.1	0.111+1.91%	111
2	0.3	0.304+1.38%	101
3	0.5	0.496+0.56%	99.2

^1^ Mean value from three measurements for each concentration

^2^ Relative standard deviation

## Conclusions

An optical fiber-based biosensing system using the optical fiber-based localized surface plasmon resonance of AuNPs for colorimetric detection of melamine in liquid milk has been investigated. It has obvious advantages over the conventional ultraviolet-visible spectrophotometer, not only because it is fast and accurate, but also is stable in the effective detection of melamine in real milk samples in practice. The experimental results demonstrate a good linear relationship between the difference of absorbance values at the wavelength of 520 nm and the concentrations of melamine in the liquid milk in the range from 0.1μM to 0.9μM and within the detection limit 33 nM. Moreover, the recovery rate of 99.2%~111% of melamine in liquid milk samples is also successfully obtained. The proposed methodology would have a huge application prospects because it also can be applied to other molecules by just selecting appropriate ligands to the to-be-detected molecules.

## Supporting information

S1 Fig UV-Visible absorption spectra of AuNPs (Blank), AuNPs+0.5μM melamine, AuNPs+2μM melamine and AuNPs+10μM melamine, indicated with dark-red- blue-pink color. (TIF)

## References

[1] Chu CY, Wang CC. Toxicity of melamine: the public health concern. J Environ Sci Heal C. 2013; 31: 342–386. 10.1080/10590501.2013.844758 24171438

[2] Dalal RP, Goldfarb DS. Melamine-related kidney stones and renal toxicity. Nat Rev Nephrol. 2011; 7: 267–274. 10.1038/nrneph.2011.24 21423252

[3] Zhu L, Gamez G, Chen HW, Chingin K, Zenobi R. Rapid detection of melamine in untreated milk and wheat gluten by ultrasound-assisted extractive electrospray ionization mass spectrometry (EESI-MS). Chem Commun. 2009; 5: 559–561. 10.1039/b818541g 19283290

[4] Filazi A, Sireli UT, Ekici H, Can HY, Karagoz A. Determination of melamine in milk and dairy products by high performance liquid chromatography. J Dairy Sci. 2012; 95: 602–608. 10.3168/jds.2011-4926 22281324

[5] Vail TM, Jones PR, Sparkman OD. Rapid and unambiguous identification of melamine in contaminated pet food based on mass spectrometry with four degrees of confirmation. J Anal Toxicol. 2007; 31: 304–312. 17725875 10.1093/jat/31.6.304

[6] Xu XM, Ren YP, Zhu Y, Cai ZX, Han JL, Huang BF, et al Direct determination of melamine in dairy products by gas chromatography/mass spectrometry with coupled column separation. Anal Chim Acta. 2009; 650: 39–43. 10.1016/j.aca.2009.04.026 19720170

[7] Filigenzi MS, Puschner B, Aston LS, Poppenga RH. Diagnostic determination of melamine and related compounds in kidney tissue by liquid chromatography/tandem mass spectrometry. J Agr Food Chem. 2008; 56: 7593–7599. 18652475 10.1021/jf801008s

[8] Wen Y, Liu H, Han P, Gao Y, Luan F, Li X. Determination of melamine in milk powder, milk and fish feed by capillary electrophoresis: a good alternative to HPLC. J Sci Food Agr. 2010; 90: 2178–2182. 20623708 10.1002/jsfa.4066

[9] Cao Q, Zhao H, Zeng LX, Wang J, Wang R, Qiu XH, et al Electrochemical determination of melamine using oligonucleotides modified gold electrodes. Talanta. 2009; 80: 484–488. 10.1016/j.talanta.2009.07.006 19836508

[10] Li Y, Xu JY, Sun CY. Chemical sensors and biosensors for the detection of melamine. RSC Adv. 2015; 5: 1125–1147.

[11] Garber EAE. Detection of melamine using commercial enzyme-linked immunosorbent assay technology. J Food Protect. 2008; 71: 590–594. 10.4315/0362-028x-71.3.590 18389705

[12] Lei HT, Su R, Haughey SA, Wang Q, Xu ZL, Yang JY, et al Development of a specifically enhanced enzyme-linked immunosorbent assay for the detection of melamine in milk. Molecules, 2011; 16: 5591–5603.

[13] Aernouts B, Van BR, Watte R, Huybrechts T, Lammertyn J, Saeys W. Visible and near-infrared bulk optical properties of raw milk. J Dairy Sci. 2015; 98: 6727–6738. 10.3168/jds.2015-9630 26210269

[14] Zhang XF, Zou MQ, Qi XH, Liu F, Zhu XH, Zhao BH. Detection of melamine in liquid milk using surface-enhanced Raman scattering spectroscopy. J Raman Spectrosc. 2010; 41: 1655–1660.

[15] Polavarapu L, Perez-Juste J, Xu QH, Liz-Marzan LM. Optical sensing of biological, chemical and ionic species through aggregation of plasmonic nanoparticles. J Mater Chem C. 2014; 2: 7460–7476.

[16] Wei H, Li BL, Li J, Wang EK, Dong SJ. Simple and sensitive aptamer-based colorimetric sensing of protein using unmodified gold nanoparticle probes. Chem Commun. 2007; 36:3735–3737. 10.1039/b707642h 17851611

[17] Sun JF, Guo L, Bao Y, Xie JW. A simple, label-free aunps-based colorimetric ultrasensitive detection of nerve agents and highly toxic organophosphate pesticide. Biosens Bioelectron. 2011; 28: 152–157. 10.1016/j.bios.2011.07.012 21803563

[18] Zhu YY, Cai YL, Zhu YB, Zheng LX, Ding JY, Quan Y, et al Highly sensitive colorimetric sensor for Hg(2+) detection based on cationic polymer/dna interaction. Biosens Bioelectron. 2015; 69:174–178. 10.1016/j.bios.2015.02.018 25727033

[19] Polavarapu L, Mourdikoudis S, Pastoriza-Santos I, Perez-Juste J. Nanocrystal engineering of noble metals and metal chalcogenides: controlling the morphology, composition and crystallinity. Cryst Eng Comm. 2015; 17: 3727–3762.

[20] Cretu V, Postica V, Mishra AK, Hoppe M, Tiginyanu I, Mishra YK, et al Synthesis, characterization and DFT studies of zinc-doped copper oxide nanocrystals for gas sensing applications. J Mater Chem A. 2016; 4: 6527–6539.

[21] Chi H, Liu BH, Guan GJ, Zhang ZP, Han MY. A simple, reliable and sensitive colorimetric visualization of melamine in milk by unmodified gold nanoparticles. *Analyst*, 2010; 135: 1070–1075. 10.1039/c000285b 20419258

[22] Wei F, Lam R, Cheng S, Lu S, Ho D, Li N. Rapid detection of melamine in whole milk mediated by unmodified gold nanoparticles. Appl Phys Lett. 2010; 96: 133702 10.1063/1.3373325 20428252 PMC2859078

[23] Kumar N, Seth R, Kumar H. Colorimetric detection of melamine in milk by citrate-stabilized gold nanoparticles. Anal Biochem. 2014; 456: 43–49. 10.1016/j.ab.2014.04.002 24727351

[24] Wang CL, Astruc D. Nanogold plasmonic photocatalysis for organic synthesis and clean energy conversion. Chem Soc Rev. 2014; 43: 7188–7216. 10.1039/c4cs00145a 25017125

[25] Hwang WS, Truong PL, Sim SJ. Size-dependent plasmonic responses of single gold nanoparticles for analysis of biorecognition. Anal Biochem. 2012; 421: 213–218. 10.1016/j.ab.2011.11.001 22146558

[26] Sepulveda B, Angelome PC, Lechuga LM, Liz-Marzan LM. LSPR-based nanobiosensors. Nanotoday. 2009; 4: 244–251.

[27] Papavlassopoulos H, Mishra YK, Kaps S, Paulowicz I, Abdelaziz R, Elbahri M, et al Toxicity of Functional Nano-Micro Zinc Oxide Tetrapods: Impact of Cell Culture Conditions, Cellular Age and Material Properties. PLoS ONE. 2014; 13: e84983. 10.1371/journal.pone.0084983 PMC3890288 24454775

[28] Jin X, Deng M, Kaps S, Zhu XW, Hölken I, Mess K, et al Study of Tetrapodal ZnO-PDMS Composites: A Comparison of Fillers Shapes in Stiffness and Hydrophobicity Improvements. PLoS ONE. 2014; 9: e84983. 25208080 10.1371/journal.pone.0106991 PMC4160218

[29] Shiohara A, Langer J, Polavarapua L, Liz-Marzan LM. Solution processed polydimethylsiloxane/gold nanostar flexible substrates for plasmonic sensing. Nanoscale. 2014; 6: 9817–9823. 10.1039/c4nr02648a 25027634

[30] Najim M, Modi G, Mishra YK, Adelung R, Singh D, Agarwalaad V. Ultra-wide bandwidth with enhanced microwave absorption of electroless Ni–P coated tetrapod-shaped ZnO nano- and microstructures. Phys Chem Chem Phys. 2015; 17: 22923–22933. 10.1039/c5cp03488d 26267361

[31] Kelly KL, Coronado E, Zhao LL, Schatz GC. The optical properties of metal nanoparticles: the influence of size, shape, and dielectric environment. J Phys Chem B. 2003; 34: 668–677.

[32] Willets KA, Van Duyne RP. Localized surface plasmon resonance spectroscopy and sensing. Annu Rev Phys Chem. 2007; 58: 267–297. 10.1146/annurev.physchem.58.032806.104607 17067281

[33] Haes AJ, Van Duyne RP. A nanoscale optical biosensor: sensitivity and selectivity of an approach based on the localized surface plasmon resonance spectroscopy of triangular silver nanoparticles. J Am Chem Soc. 2002; 124: 10596–10604. 12197762 10.1021/ja020393x

[34] Li ZJ, Zheng XJ, Zhang L, Liang RP, Li ZM, Qiu JD. Label-free colorimetric detection of biothiols utilizing SAM and un-modified Au nanoparticles. Biosens Bioelectron. 2015; 68: 668–674. 10.1016/j.bios.2015.01.062 25660511

[35] Ping H, Zhang MW, Li HK, Li SG, Chen QS, Sun CY, et al Visual detection of melamine in raw milk by label-free silver nanoparticles. Food Control. 2012; 23: 191–197.

[36] Xin JY, Zhang LX, Chen DD, Lin K., Fan HC, Wang Y, et al Colorimetric detection of melamine based on methanobactin-mediated synthesis of gold nanoparticles. Food Chem. 2015; 174: 473–479. 10.1016/j.foodchem.2014.11.098 25529708

[37] Song J, Wu FY, Wan YQ, Ma LH. Colorimetric detection of melamine in pretreated milk using silver nanoparticles functionalized with sulfanilic acid. Food Control. 2015; 50: 356–361.

